# Examining the impact of socioeconomic status, demographic characteristics, lifestyle and other risk factors on adults' cognitive functioning in developing countries: an analysis of five selected WHO SAGE Wave 1 Countries

**DOI:** 10.1186/s12939-022-01622-7

**Published:** 2022-02-25

**Authors:** Ebenezer Larnyo, Baozhen Dai, Jonathan Aseye Nutakor, Sabina Ampon-Wireko, Abigail Larnyo, Ruth Appiah

**Affiliations:** 1 Department of Health Policy and Management, School of Management, Jiangsu University, 301# Xuefu Road, Zhenjiang, 212013 Jiangsu Province China; 2 Department of Labor and Social Security, School of Public Health, Southeast University, 87# Dingjiaqiao, Nanjing, 210009 Jiangsu province China; 3 School of Management, Jiangsu University, 301# Xuefu Road, Zhenjiang, 212013 Jiangsu Province China

**Keywords:** Socioeconomic status, Cognitive functioning, Adults, Developing countries

## Abstract

**Background:**

Though extensive studies have been conducted on assessing the predictors of cognitive functioning among older adults in small community-based samples, very few studies have focused on understanding the impact of socioeconomic status (SES), demographic characteristics and other risk factors such as lifestyle and chronic diseases on the cognitive functioning among adults of all ages in a nationally representative population-based sample across low- and middle-income countries. This study, therefore, seeks to evaluate the impact of SES, demographic characteristics and risk factors on the cognitive functioning of adults across all ages in five selected developing countries.

**Methods:**

Data from 12,430 observations obtained from the WHO Study on Global AGEing and Adult Health (SAGE) Wave 1; consisting of 2,486 observations each for China, Ghana, India, the Russian Federation, and South Africa, were used for the study. A meta-regression and a five-step hierarchical linear regression were used to analyze the data, with cognitive functioning as the dependent variable. Independent variables used in this study include SES; assessed by household income and education, demographic characteristics, other risk factors such as lifestyle, self-reported memory difficulty and chronic diseases.

**Results:**

This study found that SES and lifestyle significantly predicted cognitive functioning in all the five selected countries as obtained by the pooled results of the meta-regression analysis. The hierarchical linear regression results also revealed that demographic characteristics such as age, type of residency, and self-reported memory difficulty significantly impact cognitive functioning in China, Ghana, Russia, and South Africa.

**Conclusion:**

The findings in this study provide new insights for policymakers, caregivers, parents, and individuals, especially those in developing countries, to implement policies and actions targeted at improving SES and eliminating risk factors associated with cognitive decline, as these measures could help improve the cognitive functioning among their populations.

**Supplementary Information:**

The online version contains supplementary material available at 10.1186/s12939-022-01622-7.

## Introduction

The World Health Organization (WHO) has projected that by 2025, the adult population will increase to about 5.7 billion from the 4.7 billion in 2010 [1]. As the population increases at such a fast pace, non-communicable diseases such as diabetes, heart disease, and cerebrovascular disease leading to strokes, and the phenomenon of early onset of aging-related illnesses are becoming prevalent [2]. Additionally, risk factors associated with life course, with possible consequences not only for an adults’ current wellbeing but also for their health as they advance in age, are expected to increase further the pressure on adults and the healthcare systems [3, 4]. Owing to these, all countries are likely to face major challenges related to building effective and reliable health and social systems that can cater for this demographic shift, particularly those in developing countries.

For most adults, the maintenance of independence mostly requires physically and mentally draining tasks like  managing active daily living. Such daily activities can be inhibited by declining cognitive functioning, including basic cognitive abilities like memory, learning, reasoning, and knowledge [5]. Research has shown that people who have better cognitive functioning in the early stages of their lives have better health outcomes such as improved quality of life, lower risk of disabilities, and mortality [6–9] at an older age.

While cognitive functioning is crucial in measuring intrinsic capacity [10], evidence exists of the association between SES [11, 12] and cognitive functioning [13]. An improvement in SES, childhood health, nutrition, social support systems [14, 15], havebeen identified to help improve cognitive functioning as one grows. Furthermore, studies have found strong connections between socioeconomic factors, lifestyle, conditions of health, mental health, and cognitive abilities among adults [16]. Others have also revealed that socioeconomic factors together with age [16–19], sex [19], higher educational level [16, 19–22], economic status [17, 20, 22] and residency [17] remain very crucial indicators in determining better cognitive functionality. For instance, studies have shown a significant relationship between age and several different types of cognitive measures [23–26]. A study by Stephan et al. 2014 found a positive relationship between a younger subjective age and memory self-efficacy. This relationship has consequences for maintaining cognitive functioning with advancing age [27, 28]. Another study by Murman, 2015 observed that the aging of adults tends to accelerate aging-related diseases such as dementia by increasing the rate of neuronal dysfunction, neuronal loss, and cognitive decline [29], thereby impairing their everyday functional abilities. Marital status has also been found to impact adults' mental and physical health [30–33]. Studies by Sundström et al. 2016 and Feng et al. 2014 revealed that unmarried adults have a high propensity of suffering from cognitive impairments leading to dementia than married adults [34, 35]. Previous research has also observed that rural dwellers tend to have a significantly higher prevalence of cognitive limitations than their counterparts in urban residences [36, 37]. Research on the association between sex and cognitive functioning has produced mixed results. While studies have observed that  differences in sex is correlated with cognitive functioning [38–41], others have found comparable cognitive functioning in both sexes [42, 43]. Given that these factors have implications for an adults’ cognitive functioning, it is imperative to understand them in the context of developing countries in a nationally representative manner. Aside from these factors, lifestyle risk factors like tobacco use and alcohol consumption [44] have been established to negatively impact adults’ cognitive performance . The existence of these empirical pieces of evidence only gives credence to the fact that SES, lifestyle, demographics and chronic diseases have permeative consequences on health outcomes, including neurological conditions such as cognitive functioning across an adults’ life course.

Notwithstanding the plethora of literature, only a few studies have sought to understand the impact of SES, lifestyle, demographics and chronic disease on the overall effect on cognitive abilities, especially among adults across all ages. Most of these studies have largely focused on understanding the role these predictors play on cognitive functioning only among older adults in low- and middle countries. Others have also limited their studies to small community-based samples in developing countries; hence, the reliability and validity of the results cannot be generalized to a wider population due to the several differences in both country geographic and socioeconomic indicators. These limitations suggest the need to employ more representative population-based samples to explain better the phenomenon relating to the assessment of predictors of cognitive functioning among adults (both young adults and elderly) in low- and middle-income countries.

Thus, this study seeks to address these gaps by evaluating the impact of socioeconomic status, demographic characteristics and risk factors on the cognitive functioning of adults across all ages and to further assess their contributing role in cognitive functioning disparities in five selected low- and middle-income countries, using a nationally representative population-based sample from the WHO Global Ageing and Adult Health (SAGE) Wave 1 data [45, 46].

### Methodology

#### Sample, sampling procedure, and data collection

The sample for this study was made up of adults aged 18 + in five (China, Ghana, India, Russian Federation, and South Africa) of the six Study on Global AGEing and Adult Health (SAGE) Wave 1 countries, excluding Mexico. Mexico was excluded due to substantially incomplete and missing data, especially demographic characteristics. A total of 12,430 observations were used for this study, consisting of 2,486 observations each for China, Ghana, India, the Russian Federation, and South Africa. SAGE Wave 1 is a cross-sectional study that provides baseline survey and biomarker data for nationally representative samples of adults' health and wellbeing from six low and middle-income countries (China, Ghana, India, Mexico, Russian Federation, and South Africa) [46, 47].

The data were collected via in-person structured interviews; paper and pencil interviews (PAPI) in Ghana, India, Russian Federation, and South Africa, and 50% computer-assisted personal interviews (CAPI), 50% PAPI in China [48]. The selection of samples for this study was done by implementing multistage cluster sampling strategies.

To preserve the confidentiality and anonymity of respondents, personal data identifying respondents were removed from the data across all the countries involved in the study.

### Measures

#### Outcome variable

In line with the aim of this study, cognitive functioning was selected as the outcome variable. Cognitive functioning was evaluated using a cognitive battery test, consisting of forward digit span and backward digit span, immediate recall, verbal recall, and verbal fluency [46]. This was done by allowing respondents to repeatedly read forward and backward the lists of numbers in series. These readings were then scored, with respondents who can repeat these numbers without mistakes considered to have better recall and focus. Immediate and delayed verbal recalls were used to assess respondents' memory and learning ability . A 10-wordlist of animals was read out to respondents to listen carefully and  remember as many words as possible. This was done iteratively for three trials, with better recall scores indicating higher learning and memory ability. These scores were standardized and added up to evaluate the cognitive functioning of respondents, with higher standardized scores depicting better cognitive functioning [46].

#### Independent variables

The independent variable used to predict cognitive functioning in this study included socioeconomic status which was evaluated using the household income quantiles and the educational level of respondents, type of residence, sex, marital status, lifestyle, chronic diseases, and self-rated memory difficulty. Studies have found education and income to be among the key predictors of mental wellbeing among adults [49–51]. More significantly, these studies have consistently identified a strong positive relationship between higher education and better cognition [49, 50, 52, 53]. Thus, the inclusion of these indicators is essential in understanding adults’ cognitive health. In this study, household income was grouped into five income levels; lowest, low, moderate, higher, and highest income quantiles, while respondents' educational level was segregated into no formal education, less than primary school, the primary school completed, the secondary school completed, high school (or equivalent) completed, college/university/postgraduate degree completed. The secondary school completed and high school (or equivalent) completed were not merged since the five selected countries had different educational systems. This study categorized age into seven bands; 18–24, 25–34, 35–44, 45–54, 55–64, 65–74, and 75 + , type of residence; rural and urban, sex; male and female, and marital status; never married, currently married, cohabiting, separated/divorced, widowed. Lifestyle was assessed using alcohol and tobacco consumption, the number of fruits and vegetables consumed per day, while chronic diseases were evaluated using stroke, diabetes, chronic lung disease, depression, anxiety and hypertension. Lastly, respondents’ self-reported memory difficulty was rated as very bad, bad, moderate, good and very good.

### Data analysis

The data analysis for this study included a reliability test for the outcome variable, descriptive analysis, a meta-regression and hierarchical linear regression using STATA SE version 15.0 (Stata Corp, college station, Tx) and Intellectus statistics (Intellectus Statistics [Online computer software], 2020). The descriptive analysis was performed on the nominal variables of the sample demographic by assessing their frequencies and percentages. Summary statistics were also computed using averages, skewness, median, and kurtosis [54]. The likely score of heterogeneity was evaluated via a meta-regression. Using the Higgins' I^2^ statistic, the homogeneity of cognitive functioning across the selected countries was assessed. An I^2^ score above 50% suggests a high heterogeneity [55]. The 95% Confidence Intervals (CI) of cognitive functioning was examined using the Dersimonian and Laird random-effect model. A graphical representation of the estimates is presented in a forest plot. The hierarchical linear regression was conducted to assess the level of contribution of the various variables in predicting cognitive functioning and also to determine which of the variables explains significantly more variance of the dependent variable.

Finally, to account for the complexity in the survey data, the survey datasets (svyset) command was run in Stata using the sampling weights, primary sampling units (PSU), and strata variables for the in-country samples and in the multi-country data set.

## Results

### Reliability test for items used to assess cognitive functioning

Results for the reliability test for the items used to evaluate cognitive functioning revealed that Cronbach alpha values were mostly within the moderate thresholds [56, 57] for almost all the selected countries (see supplementary material Table A1).

### Sample demographic characteristics

Sample demographic characteristics consisting of frequencies and percentages for the variables used for this study are presented in Table 1. The most frequently observed residence category for China, Ghana, and India was Rural, while for Russia and South Africa was Urban. Regarding sex, the female category was the most frequently observed category for Ghana, Russia, and South Africa, while that for China and India was male. The majority of respondents for all the selected countries were between ages 55 to 64, while the majority were currently married at the time of the study for all countries.

**Table 1:** Frequency and percentage table for sample socio-demographic characteristics

Variables	Countries									
	**China**		**Ghana**		**India**		**Russia**		**South Africa**	
	*n*	*%*	*n*	*%*	*n*	*%*	*n*	*%*	*n*	*%*
**Residence**										
Urban	947	38.09	806	32.42	473	19.03	1764	70.96	1697	68.26
Rural	1539	61.91	1680	67.58	2013	80.97	722	29.04	789	31.74
**Sex**										
Male	2379	95.70	1158	46.58	1478	59.45	879	35.36	1052	42.32
Female	107	4.30	1328	53.42	1008	40.55	1607	64.64	1434	57.68
**Age**										
18–24	12	0.48	14	0.56	50	2.01	19	0.76	39	1.57
25–34	50	2.01	58	2.33	147	5.91	72	2.90	50	2.01
35–44	134	5.39	97	3.90	288	11.58	101	4.06	91	3.66
45–54	580	23.33	429	17.26	513	20.64	505	20.31	607	24.42
55–64	962	38.70	693	27.88	729	29.32	712	28.64	833	33.51
65–74	507	20.39	668	26.87	528	21.24	638	25.66	583	23.45
75 +	241	9.69	527	21.20	231	9.29	439	17.66	283	11.38
**Marital Status**										
Never Married	56	2.25	50	2.01	58	2.33	97	3.90	401	16.13
Currently Married	2218	89.22	1282	51.57	1924	77.39	1399	56.28	1161	46.70
Cohabiting	3	0.12	23	0.93	2	0.08	77	3.10	135	5.43
Separated/Divorced	62	2.49	360	14.48	19	0.76	220	8.85	156	6.28
Widowed	147	5.91	771	31.01	483	19.43	693	27.88	633	25.46
**Educational Level**										
No Formal Education	328	13.19	1597	64.24	1365	54.91	23	0.93	565	22.73
Less than Primary School	497	19.99	327	13.15	301	12.11	47	1.89	514	20.68
Primary School Completed	586	23.57	228	9.17	377	15.16	174	7.00	591	23.77
Secondary School Completed	626	25.18	80	3.22	233	9.37	442	17.78	435	17.50
High School (or equivalent) Completed	349	14.04	233	9.37	149	5.99	1339	53.86	248	9.98
College/University/Postgraduate Completed	100	4.02	21	0.84	61	2.45	461	18.54	133	5.35
**Income Quintiles**										
Lowest	465	18.70	625	25.14	615	24.74	447	17.98	447	17.98
Low	511	20.56	538	21.64	572	23.01	473	19.03	486	19.55
Moderate	503	20.23	558	22.64	517	20.80	469	18.87	493	19.83
Higher	570	22.93	446	17.94	449	18.06	511	20.56	531	21.36
Highest	437	17.58	319	12.83	333	13.40	586	23.57	529	21.28
**Self-Reported Memory Difficulty**										
Very Bad	32	1.29	9	0.36	20	0.80	4	0.16	11	0.44
Bad	690	27.76	117	4.71	302	12.15	223	8.79	212	8.53
Moderate	1141	45.90	1124	45.21	1226	49.32	1215	48.87	918	36.93
Good	561	22.57	1095	44.05	874	35.16	981	39.46	1194	48.03
Very Good	62	2.49	141	5.67	64	2.57	63	2.53	151	6.07
**Chronic Diseases**										
**Stroke**										
Yes	80	3.22	68	2.74	53	2.13	122	4.91	93	3.74
No	2406	96.78	2418	97.26	2433	97.87	2364	95.09	2393	96.26
**Diabetes**										
Yes	101	4.06	65	2.61	122	4.91	195	7.84	237	9.53
No	2385	95.94	2421	97.39	2364	95.09	2291	92.16	2249	90.47
**Chronic Lung Disease**										
Yes	259	10.42	16	0.64	119	4.79	433	17.42	46	1.85
No	2227	89.58	2470	99.36	2367	95.21	2053	82.58	2440	98.15
**Depression**										
Yes	3	0.12	42	1.69	155	6.23	97	3.90	117	4.71
No	2483	99.88	2444	98.31	2331	93.77	2389	96.10	2369	95.29
**Anxiety**										
More Anxious	177	7.12	708	28.48	513	20.64	479	19.27	320	12.87
Same Level of Anxiety	1821	73.25	1190	47.87	1216	48.91	1700	68.38	1435	57.72
Less Anxious	488	19.63	588	23.65	757	30.45	307	12.35	731	29.40
**Hypertension**										
Yes	498	20.03	258	10.38	339	13.64	1339	53.86	712	28.64
No	1988	79.97	2228	89.38	2147	86.36	1147	46.14	1774	71.36
**Lifestyle**										
**Alcohol Use: Ever used alcohol?**										
Yes	2479	99.72	1375	55.31	660	26.55	1849	74.38	674	27.11
No, never	7	0.28	1111	44.69	1826	73.45	637	25.62	1812	72.89
**Tobacco Use: Ever used tobacco?**										
Yes	2483	99.88	606	24.38	2486	100	758	30.49	858	34.51
No	3	0.12	1880	75.62	-	-	1728	69.51	1628	65.49
**Fruits: How many servings of fruits per day?**										
0–5	2382	95.82	2379	95.70	2474	99.52	2448	98.47	2467	99.24
6–10	99	3.98	105	4.22	11	0.44	37	1.49	18	0.72
11–15	-	-	1	0.04	1	0.04	1	0.04	1	0.04
16 +	5	0.20	1	0.04	-	-	-	-	-	-
**Vegetables: How many servings of vegetables?**										
0–5	1048	42.16	2473	99.48	2456	98.79	2441	98.19	2423	97.47
6–10	1102	44.33	11	0.44	30	1.21	43	1.73	60	99.88
11–15	158	6.35	2	0.08	-	-	2	0.08	3	0.12
16 +	178	7.16	-	-	-	-	-	-	-	-

*Note.* Due to rounding errors, percentages may not equal 100%; '-' indicates the statistic is undefined due to constant data, insufficient sample size, or no response

The most frequently observed category for the level of education in China was respondents who had completed secondary school, Ghana and India those who had no formal education, Russia those who had completed high school (or equivalent), and those who completed primary school for South Africa.

Tobacco consumption was highest among respondents from China and India, while alcohol consumption was prevalent in China, Ghana, and Russia. In the most frequently observed category, the number of fruits served per day was 0–5 for all countries. The majority of respondents in China and South Africa consumed between 6–10 vegetables per day, while their counterparts in Ghana, India, and Russia averaged between 0–5 vegetables served per day. The most frequently observed category of self-reported memory difficulty was moderate for China, Ghana, India, and Russia, while South Africa was Good (*n* = 1194, 48%). Supplementary material Table A2 shows the summary statistics for age, education, cognitive functioning and income (please see supplementary material Table A2).

### Meta-regression analysis

**Fig. 1:**
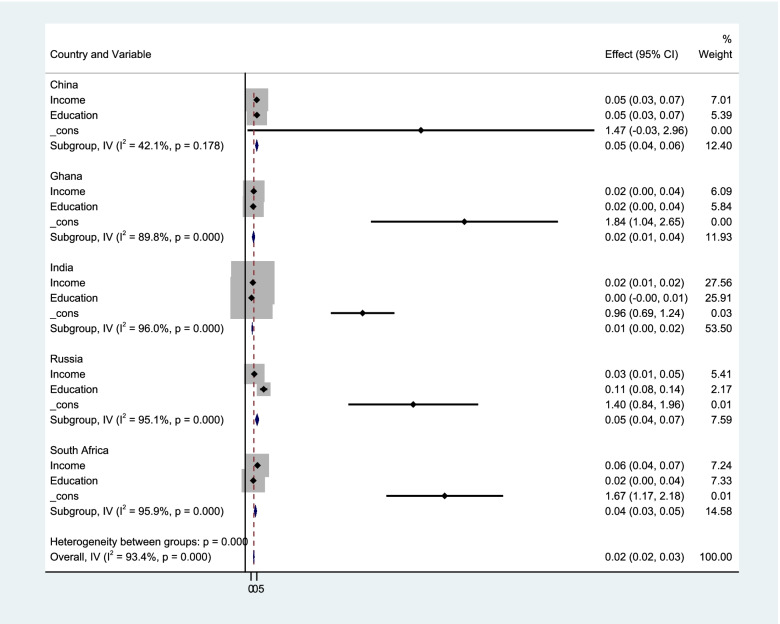
Forest plot of the impact of socioeconomic status on cognitive functioning among the five selected countries

Figure 1 shows a forest plot of the impact of SES on cognitive functioning in the selected countries. The meta-regression analysis revealed a pooled cognitive functioning of 2% (95% CI: 0.02–0.03, I^2^ = 93.4%, *p* < 0.001). This indicates that on average, a one-unit increase in the SES will increase the value of cognitive functioning by 0.02, giving credence to the fact that there is strong evidence that SES does improve cognitive functioning. Similar trend was also observed for lifestyle (0.02 (95% CI: 0.01, 0.04, I^2^ = 89.1%, *p* < 0.001). A pooled cognitive functioning of -0.01 (95% CI: -0.02,-0.01, I^2^ = 92.0%, *p* < 0.001), -0.03 (95% CI: -0.04,-0.02, I^2^ = 95.2%, *p* < 0.001), -0.00 (95% CI: -0.01, 0.01, I^2^ = 84.6%, *p* < 0.001) was observed for demographic characteristics, self-reported memory difficulty and chronic diseases, respectively (please see supplementary material Figure A1, A2, A3 and A4).

### Hierarchical linear regression

A five-step hierarchical linear regression was conducted with cognitive functioning as the dependent variable. The hierarchical regression analysis results consist of model comparisons and a model interpretation based on an alpha of 0.05. Each step in the hierarchical regression was compared to the previous step using *F*-tests.

### Model comparison

The *F-test* for Step 1 was significant for China, Ghana, India, Russia, and South Africa, indicating that adding sex, age, residence, and marital status explained an additional 6.92%, 2.99%, 1.47%, 9.86%, and 2.32%, respectively, of the variation in cognitive functioning in the five selected countries. The *F-test* for step 2 was significant for China, Ghana, Russia, and South Africa, but not India. This suggests that adding self-reported memory difficulty explained an additional 0.91%, 0.69%, 0.87%, and 2.26% of the variation in cognitive functioning in China, Ghana, Russia, and South Africa, respectively. Adding tobacco use, alcohol use, vegetable and fruit servings per day yielded a significant *F-test* for step 3 for China, Ghana, Russia, and South Africa; however, this was not the case for India. Furthermore, introducing chronic diseases in step 4 produced significant *F-test* for China, Ghana, and Russia, but not for India and South Africa. Finally, the *F-test* for step 5 was significant for all five countries, indicating that adding education and income explained an additional 2.49%, 0.49%, 1.19%, 2.17%, and 2.50% of the variation in cognitive functioning for China, Ghana, India, Russia, and South Africa, respectively, as shown in Table 2.

**Table 2:** Model comparisons for variables predicting cognitive functioning

China										Ghana										India									
**Model**	***R***^**2**^		***df***_**mod**_	***df***_**res**_	***F***		***p***		**Δ*R***^**2**^	***R***^**2**^		***df***_**mod**_		***df***_**res**_	***F***		***p***		**Δ*R***^**2**^	***R***^**2**^		***df***_**mod**_		***df***_**res**_	***F***		***p***		**Δ*R***^**2**^
Step 1	.07		4	2481	46.14		***		.07	.03		4		2481	19.09		***		.03	.01		4		2481	9.26		***		.01
Step 2	.08		1	2480	24.59		***		.01	.04		1		2480	17.85		***		.01	.02		1		2480	1.56		.211		.00
Step 3	.09		4	2476	5.51		***		.01	.05		4		2476	7.87		***		.01	.02		3		2477	1.91		.126		.00
Step 4	.09		6	2470	2.73		**		.01	.07		6		2470	7.93		***		.02	.02		6		2471	0.70		.647		.00
Step 5	.12		2	2468	34.81		***		.02	.07		2		2468	6.53		***		.00	.03		2		2469	15.13		***		.01
**Russia**																**South Africa**													
**Model**		***R***^**2**^		***df***_**mod**_		***df***_**res**_		***F***			***p***		**Δ*R***^**2**^			***R***^**2**^		***df***_**mod**_			***df***_**res**_		***F***			***p***		**Δ*R***^**2**^	
Step 1		.10		4		2481		67.87			***		.10			.02		4			2481		14.72			***		.02	
Step 2		.11		1		2480		24.14			***		.01			.05		1			2480		58.63			***		.02	
Step 3		.11		4		2476		2.96			**		.00			.06		4			2476		7.05			***		.01	
Step 4		.12		6		2470		2.34			**		.01			.06		6			2470		1.99			.064		.00	
Step 5		.14		2		2468		31.15			***		.02			.09		2			2468		33.73			***		.02	

*Note.* Each Step was compared to the previous model in the hierarchical regression analysis. *** p < .01, ** p < *.05*

### Model interpretation

Sex (*B* = -0.14, *t* (2468) = -2.47, *p* = 0.014.), age (*B* = -0.06, *t* (2468) = -4.94, *p* < 0.001), residence (*B* = -0.10, *t* (2468) = -3.78, *p* < 0.001) and self-reported memory difficulty (*B* = -0.06, *t* (2468) = -4.10, *p* < 0.001), significantly predicted cognitive functioning in China. These indicate that on average, a differences in sex, a one-unit increase of age, a change in the type of residence and a one-unit increase of self-reported memory difficulty will decrease the value of cognitive functioning by 0.14, 0.06, 0.10 and 0.06 units respectively. Also, vegetables per day (*B* = 0.06, *t* (2468) = 4.55, *p* < 0.001), anxiety (*B* = 0.05, *t* (2468) = 2.24, *p* = 0.025), stroke (*B* = 0.14, *t* (2468) = 2.15, *p* = 0.032), education (*B* = 0.05, *t* (2468) = 5.06, *p* < 0.001), and income (*B* = 0.05, *t* (2468) = 5.61, *p* < 0.001) significantly predicted cognitive functioning in China. These indicate that on average, a one-unit increase in vegetable consumption per day, anxiety level, stroke, education, and income will increase the value of cognitive functioning by 0.05, 0.06, 0.05, 0.14 and 0.05 units respectively. However, marital status, tobacco use, alcohol use, fruits per day, chronic lung disease, hypertension, diabetes and depression did not significantly predict cognitive functioning, suggesting that, a one-unit increase or change in any of these variables do not have significant effect on cognitive functioning in China.

For Ghana, age (*B* = *-0.03, t (2468)* = *-2.74, p* = *0.006*), self-reported memory difficulty (*B* = *-0.06, t (2468)* = *-3.42, p* < *0.001*) and anxiety (*B* = -0.10, *t* (2468) = -5.89, *p* < 0.001), significantly predicted cognitive functioning. These indicate that on average, a one-unit increase of age, self-reported memory difficulty and anxiety will decrease the value of cognitive functioning by 0.03, 0.06 and 0.10 units respectively. Also, residence (*B* = *0.10, t (2468)* = *3.70, p* < *0.001*, alcohol (*B* = *0.08, t (2468)* = *3.08, p* = *0.002*), fruits per day (*B* = *0.21, t (2468)* = *3.78, p* < *0.001*), anxiety (*B* = *-0.10, t (2468)* = *-5.89, p* < *0.001*), stroke (*B* = *0.23, t (2468)* = *3.14, p* = *0.002*)), education (*B* = *0.02, t (2468)* = *1.98, p* = *0.048*) and income (*B* = *0.02, t (2468)* = *2.49, p* = *0.013*) significantly predicted cognitive functioning in Ghana. These indicate that on average, a change in residence, a one-unit increase in the level of education, alcohol use, fruits per day, anxiety, stroke and income will increase the value of cognitive functioning by 0.10, 0.02, 0.08, 0.21, 0.10, 0.23 and 0.02 units respectively. However, sex, marital status, tobacco use, vegetables per day, chronic lung disease, hypertension, diabetes and depression did not significantly predict cognitive functioning. Based on these samples, a one-unit increase or change in any of these variables do not have significant effect on cognitive functioning in Ghana.

Sex *(B* = -0.21, *t* (2469) = -2.89, *p* = 0.004) and marital status (*B* = *-0.01, t (2469)* = *-2.81, p* = *0.005*) significantly predicted cognitive functioning in India. These indicate that, on average, a change in marital status and difference in sex will decrease the value of cognitive functioning by 0.21 and 0.10 units. Also, residence (*B* = *0.03, t (2469)* = *2.07, p* = *0.038*), education (*B* = 0.06, *t*(2469) = 2.63, *p* = 0.009) and income (*B* = *0.02, t (2468)* = *2.49, p* = *0.013*) significantly predicted cognitive functioning, indicating that on average, a change in the type of residence, educational level and a one-unit increase of income will increase the value of cognitive functioning by 0.03, 0.06 and 0.02 units respectively in India. However, age, self-reported memory difficulty, alcohol use, vegetables per day, fruits per day, chronic lung disease, hypertension, diabetes, anxiety, stroke, and depression did not significantly predict cognitive functioning.

For Russia, age (*B* = *-0.09, t (2468)* = *-7.25, p* < *0.00*), self-reported memory difficulty (*B* = *-0.07, t (2468)* = *-3.04, p* = *0.002*), alcohol use (*B* = -0.06, *t* (2468) = -2.02, *p* = 0.043), tobacco use (*B* = -0.08, *t* (2468) = -2.19, *p* = 0.029), and residence (*B* = *-0.12, t (2468)* = *-3.90, p* < *0.001*) significantly predicted cognitive functioning. These indicate that on average, a one-unit increase of age, self-reported memory difficulty, alcohol use, tobacco use, and a change in residence, will decrease the value of cognitive functioning by 0.09, 0.12, 0.07, 0.06 and 0.08 units respectively. Also, sex (*B* = 0.09, *t* (2468) = 2.56, *p* = 0.011), stroke (*B* = *0.14, t (2468)* = *2.20, p* = *0.028*), depression (*B* = *0.16, t (2468)* = *2.30, p* = *0.021,* education (*B* = *0.11, t (2468)* = *6.92, p* < *0.001*), and income (*B* = *0.03, t (2468)* = *2.83, p* = *0.005*) significantly predicted cognitive functioning in Russia. These indicate that on average, a change in the category of sex, a one-unit increase in stroke, depression, level of education and income will increase the value of cognitive functioning by 0.09, 0.14, 0.16, 0.11 and 0.03 units respectively. However, marital status, fruits per day, vegetables per day, chronic lung disease, hypertension, diabetes, anxiety and depression did not significantly predict cognitive functioning, suggesting that, a one-unit increase or change in any of these variables do not have significant effect on cognitive functioning.

Age (*B* = *-0.04, t (2468)* = *-4.04, p* < *0.001*) and self-reported memory difficulty (*B* = *-0.09, t (2468)* = *-5.92, p* < *0.001*), significantly predicted cognitive functioning in South Africa. These indicate that on average, a one-unit increase in age and self-reported memory difficulty will decrease the value of cognitive functioning by 0.04 and 0.09 units respectively. Further, residence (*B* = *0.11, t (2468)* = *4.70, p* < *0.001*), vegetables per day (*B* = *0.27, t (2468)* = *4.35, p* < *0.001*), education (*B* = *0.02, t (2468)* = *2.28, p* = *0.023*) and income (*B* = *0.06, t (2468)* = *6.44, p* < *0.001*) significantly predicted cognitive functioning, indicating that on average, a change in residence, a one-unit increase in vegetable consumption per day, educational level, and income will increase the value of cognitive functioning by 0.11, 0.27, 0.02, and 0.06 units respectively in South Africa. However, sex, marital status, alcohol use, tobacco use, fruits per day, chronic lung disease, hypertension, diabetes, anxiety, stroke and depression did not significantly predict cognitive functioning. Based on these samples, a one-unit increase in any of these variables do not have significant effect on cognitive functioning. The result for each regression is shown in Table 3.

**Table 3:** Summary of hierarchical regression analysis for variables predicting cognitive functioning in the five selected countries

	***China***									***Ghana***								
**Variable**	***B***	***SE***		**95% CI**		**β**	***t***		***p***	***B***	***SE***		**95% CI**		**β**	***t***		***p***
**Step 1**																		
*(Intercept)*	2.88	0.09		[2.71, 3.06]		0.00	32.20		***	2.10	0.08		[1.95, 2.25]		0.00	27.30		***
Sex	-0.20	0.06		[-0.31, -0.09]		-0.07	-3.53		***	-0.06	0.03		[-0.12, -0.00]		-0.05	-2.11		**
Age	-0.10	0.01		[-0.12, -0.08]		-0.20	-10.07		***	-0.05	0.010		[-0.07, -0.03]		-0.11	-5.18		***
Residence	-0.19	0.02		[-0.24, -0.14]		-0.16	-8.06		***	0.07	0.03		[0.02, 0.12]		0.05	2.70		**
Marital Status	-0.02	0.02		[-0.05, 0.01]		-0.02	-1.05		.292	-0.02	0.01		[-0.05, -0.00]		-0.06	-2.25		**
**Step 2**																		
*(Intercept)*	2.99	0.09		[2.81, 3.17]		0.00	32.60		***	2.21	0.08		[2.05, 2.36]		0.00	27.35		***
Sex	-0.19	0.06		[-0.30, -0.08]		-0.07	-3.33		***	-0.05	0.03		[-0.11, 0.01]		-0.04	-1.67		.096
Age	-0.09	0.01		[-0.11, -0.07]		-0.17	-8.23		***	-0.04	0.01		[-0.06, -0.02]		-0.09	-4.14		***
Residence	-0.17	0.02		[-0.22, -0.13]		-0.14	-7.31		***	0.07	0.03		[0.02, 0.12]		0.06	2.85		**
Marital Status	-0.02	0.01		[-0.05, 0.01]		-0.02	-1.16		.248	-0.02	0.01		[-0.04, -0.00]		-0.05	-2.10		**
Self-Reported Memory Difficulty	-0.07	0.01		[-0.10, -0.04]		-0.10	-4.96		***	-0.07	0.02		[-0.11, -0.04]		-0.09	-4.23		***
**Step 3**																		
*(Intercept)*	2.46	0.37		[1.73, 3.18]		0.00	6.66		***	2.12	0.17		[1.78, 2.45]		0.00	12.38		***
Sex	-0.19	0.06		[-0.30, -0.08]		-0.07	-3.37		***	-0.07	0.03		[-0.13, -0.01]		-0.06	-2.27		**
Age	-0.09	0.01		[-0.11, -0.07]		-0.17	-8.17		***	-0.04	0.01		[-0.06, -0.02]		-0.09	-4.07		***
Residence	-0.19	0.02		[-0.24, -0.14]		-0.16	-7.96		***	0.08	0.03		[0.03, 0.13]		0.06	3.23		***
Marital Status	-0.02	0.01		[-0.05, 0.01]		-0.02	-1.20		.229	-0.02	0.01		[-0.04, -0.00]		-0.05	-2.04		**
Self-Reported Memory Difficulty	-0.08	0.01		[-0.11, -0.05]		-0.11	-5.44		***	-0.07	0.02		[-0.10, -0.03]		-0.08	-3.95		***
Tobacco Use	0.33	0.33		[-0.33, 0.98]		0.02	0.98		.325	-0.01	0.03		[-0.07, 0.05]		-0.00	-0.20		.844
Alcohol Use	0.15	0.22		[-0.28, 0.57]		0.01	0.67		.503	0.09	0.02		[0.04, 0.13]		0.07	3.48		***
Vegetables Per Day	0.06	0.01		[0.03, 0.09]		0.09	4.47		***	-0.26	0.13		[-0.52, 0.01]		-0.04	-1.90		.058
Fruits Per Day	0.00	0.05		[-0.09, 0.09]		0.00	0.01		.988	0.22	0.06		[0.11, 0.33]		0.08	3.94		***
**Step 4**																		
*(Intercept)*	1.93	0.77		[0.42, 3.44]		0.00	2.51		**	2.08	0.41		[1.28, 2.88]		0.00	5.11		***
Sex	-0.19	0.06		[-0.30, -0.08]		-0.07	-3.40		***	-0.07	0.03		[-0.13, -0.01]		-0.06	-2.26		**
Age	-0.08	0.01		[-0.11, -0.06]		-0.16	-7.76		***	-0.03	0.010		[-0.05, -0.01]		-0.07	-3.46		***
Residence	-0.19	0.02		[-0.24, -0.14]		-0.16	-7.78		***	0.07	0.03		[0.02, 0.12]		0.06	2.88		**
Marital Status	-0.02	0.01		[-0.05, 0.01]		-0.02	-1.18		.239	-0.02	0.01		[-0.04, -0.00]		-0.05	-2.00		**
Self-Reported Memory Difficulty	-0.07	0.01		[-0.10, -0.05]		-0.10	-5.02		***	-0.06	0.02		[-0.10, -0.03]		-0.07	-3.47		***
Tobacco Use	0.35	0.33		[-0.30, 1.01]		0.02	1.06		.291	-0.01	0.03		[-0.07, 0.05]		-0.01	-0.30		.762
Alcohol Use	0.14	0.22		[-0.29, 0.56]		0.01	0.63		.531	0.08	0.02		[0.03, 0.13]		0.06	3.13		**
Vegetables Per Day	0.06	0.01		[0.03, 0.09]		0.09	4.45		***	-0.25	0.13		[-0.51, 0.01]		-0.04	-1.86		.062
Fruits Per Day	-0.01	0.05		[-0.10, 0.09]		-0.01	-0.03		.977	0.20	0.05		[0.09, 0.31]		0.07	3.63		***
Chronic Lung Disease	0.01	0.04		[-0.06, 0.08]		0.01	0.27		.790	0.01	0.14		[-0.27, 0.30]		0.00	0.09		.931
Anxiety	0.07	0.02		[0.03, 0.12]		0.06	3.13		**	-0.10	0.02		[-0.13, -0.06]		-0.12	-5.91		***
Hypertension	0.00	0.03		[-0.05, 0.06]		0.00	0.13		.899	-0.05	0.04		[-0.12, 0.03]		-0.02	-1.14		.254
Diabetes	0.00	0.06		[-0.11, 0.12]		0.00	0.04		.967	0.04	0.07		[-0.10, 0.19]		0.01	0.56		.578
Depression	0.01	0.32		[-0.63, 0.64]		0.00	0.02		.987	-0.12	0.09		[-0.30, 0.06]		-0.03	-1.31		.191
Stroke	0.15	0.07		[0.02, 0.28]		0.05	2.30		**	0.22	0.07		[0.08, 0.36]		0.06	3.02		**
**Step 5**																		
*(Intercept)*	1.47	0.76		[-0.03, 2.96]		0.00	1.92		.055	1.84	0.41		[1.04, 2.65]		0.00	4.48		***
Sex	-0.14	0.06		[-0.25, -0.03]		-0.05	-2.47		***	-0.05	0.03		[-0.11, 0.01]		-0.04	-1.61		.107
Age	-0.06	0.01		[-0.08, -0.03]		-0.11	-4.94		***	-0.03	0.01		[-0.05, -0.01]		-0.06	-2.74		**
Residence	-0.10	0.03		[-0.16, -0.05]		-0.08	-3.78		***	0.10	0.03		[0.05, 0.15]		0.08	3.70		**
Marital Status	-0.01	0.01		[-0.04, 0.02]		-0.01	-0.61		.544	-0.02	0.01		[-0.04, 0.00]		-0.04	-1.75		.080
Self-Reported Memory Difficulty	-0.06	0.01		[-0.09, -0.03]		-0.08	-4.10		***	-0.06	0.02		[-0.09, -0.03]		-0.07	-3.42		***
Tobacco Use	0.36	0.33		[-0.29, 1.00]		0.02	1.08		.278	-0.02	0.03		[-0.08, 0.04]		-0.02	-0.76		.447
Alcohol Use	0.09	0.21		[-0.33, 0.51]		0.01	0.43		.667	0.08	0.02		[0.03, 0.12]		0.06	3.08		**
Vegetables Per Day	0.06	0.01		[0.03, 0.09]		0.09	4.55		***	-0.26	0.13		[-0.52, 0.00]		-0.04	-1.95		**
Fruits Per Day	-0.03	0.05		[-0.13, 0.06]		-0.01	-0.71		.476	0.21	0.05		[0.10, 0.31]		0.07	3.78		***
Chronic Lung Disease	0.00	0.04		[-0.07, 0.07]		0.001	0.05		.958	0.00	0.14		[-0.28, 0.28]		0.00007	0.00		.997
Anxiety	0.05	0.02		[0.01, 0.09]		0.04	2.24		**	-0.10	0.02		[-0.13, -0.06]		-0.12	-5.89		***
Hypertension	0.02	0.03		[-0.04, 0.08]		0.01	0.65		.514	-0.03	0.04		[-0.11, 0.05]		-0.01	-0.70		.485
Diabetes	0.02	0.06		[-0.09, 0.14]		0.007	0.38		.706	0.06	0.07		[-0.09, 0.20]		0.02	0.81		.420
Depression	-0.05	0.32		[-0.68, 0.58]		-0.003	-0.15		.879	-0.11	0.09		[-0.29, 0.06]		-0.02	-1.27		.204
Stroke	0.14	0.06		[0.01, 0.26]		0.04	2.15		**	0.23	0.07		[0.09, 0.37]		0.06	3.14		**
Education	0.05	0.01		[0.03, 0.07]		0.12	5.06		***	0.02	0.01		[0.00, 0.04]		0.04	1.98		**
Income Quintile	0.05	0.01		[0.03, 0.07]		0.12	5.61		***	0.02	0.01		[0.01, 0.04]		0.05	2.49		**
	***India***									***Russia***								
**Variable**	***B***	***SE***		**95% CI**		**β**	***t***		***p***	***B***	***SE***		**95% CI**		**β**	***t***		***p***
**Step 1**																		
*(Intercept)*	8.88	0.19		[8.51, 9.25]		0.00	46.73		***	2.81	0.09		[2.65, 2.98]		0.00	32.97		***
Sex	-0.21	0.06		[-0.33, -0.08]		-0.07	-3.27		***	-0.00	0.03		[-0.06, 0.06]		-0.00	-0.03		.980
Age	-0.02	0.02		[-0.07, 0.02]		-0.02	-1.13		.258	-0.16	0.01		[-0.18, -0.13]		-0.28	-13.62		***
Residence	0.20	0.07		[0.06, 0.35]		0.05	2.76		**	-0.17	0.03		[-0.23, -0.11]		-0.11	-5.61		***
Marital Status	-0.07	0.03		[-0.12, -0.02]		-0.06	-2.63		**	-0.02	0.01		[-0.05, -0.00]		-0.05	-2.22		**
**Step 2**																		
*(Intercept)*	8.81	0.20		[8.42, 9.20]		0.00	44.18		***	2.94	0.09		[2.77, 3.12]		0.00	33.08		***
Sex	-0.22	0.06		[-0.35, -0.10]		-0.08	-3.45		***	0.01	0.03		[-0.05, 0.07]		0.01	0.24		.812
Age	-0.03	0.02		[-0.08, 0.01]		-0.03	-1.39		.164	-0.13	0.01		[-0.16, -0.11]		-0.24	-10.93		***
Residence	0.19	0.07		[0.05, 0.34]		0.05	2.65		**	-0.15	0.03		[-0.21, -0.09]		-0.10	-5.10		***
Marital Status	-0.07	0.03		[-0.12, -0.02]		-0.06	-2.63		**	-0.02	0.01		[-0.05, -0.00]		-0.05	-2.13		**
Self-Reported Memory Difficulty	0.05	0.04		[-0.03, 0.13]		0.03	1.25		.211	-0.11	0.02		[-0.15, -0.06]		-0.10	-4.91		***
**Step 3**																		
*(Intercept)*	8.46	0.46		[7.57, 9.36]		0.00	18.50		***	2.93	0.15		[2.64, 3.22]		0.00	19.79		***
Sex	-0.27	0.07		[-0.40, -0.14]		-0.09	-3.95		***	0.06	0.04		[-0.02, 0.13]		0.04	1.52		.130
Age	-0.03	0.02		[-0.08, 0.010]		-0.03	-1.54		.124	-0.13	0.01		[-0.15, -0.10]		-0.23	-10.41		***
Residence	0.20	0.07		[0.05, 0.34]		0.05	2.69		**	-0.15	0.03		[-0.21, -0.09]		-0.10	-4.96		***
Marital Status	-0.07	0.03		[-0.12, -0.02]		-0.06	-2.57		**	-0.02	0.01		[-0.05, -0.00]		-0.05	-2.10		**
Self-Reported Memory Difficulty	0.05	0.04		[-0.03, 0.13]		0.03	1.20		.229	-0.11	0.02		[-0.15, -0.07]		-0.10	-4.99		***
Tobacco Use										-0.06	0.04		[-0.13, 0.02]		-0.04	-1.53		.126
Alcohol Use	0.15	0.07		[0.01, 0.29]		0.05	2.16		**	-0.08	0.03		[-0.15, -0.02]		-0.05	-2.55		**
Vegetables Per Day	0.28	0.27		[-0.26, 0.82]		0.02	1.02		.307	0.02	0.11		[-0.20, 0.24]		0.00	0.20		.841
Fruits Per Day	-0.12	0.39		[-0.88, 0.63]		-0.01	-0.32		.752	0.08	0.12		[-0.16, 0.32]		0.01	0.66		.507
**Step 4**																		
*(Intercept)*	8.34	0.74		[6.89, 9.80]		0.00	11.26		***	2.23	0.27		[1.70, 2.76]		0.00	8.30		***
Sex	-0.29	0.07		[-0.42, -0.15]		-0.10	-4.13		***	0.07	0.04		[-0.00, 0.14]		0.05	1.90		.058
Age	-0.04	0.02		[-0.08, 0.01]		-0.04	-1.65		.099	-0.12	0.01		[-0.15, -0.10]		-0.22	-9.58		***
Residence	0.20	0.07		[0.06, 0.35]		0.06	2.75		**	-0.15	0.03		[-0.21, -0.09]		-0.10	-5.04		***
Marital Status	-0.07	0.03		[-0.12, -0.01]		-0.06	-2.52		**	-0.02	0.01		[-0.05, -0.00]		-0.05	-2.18		.029
Self-Reported Memory Difficulty	0.04	0.04		[-0.04, 0.13]		0.02	1.09		.276	-0.09	0.02		[-0.14, -0.05]		-0.09	-4.31		***
Tobacco Use										-0.06	0.04		[-0.14, 0.01]		-0.04	-1.64		.102
Alcohol Use	0.15	0.07		[0.01, 0.29]		0.05	2.17		**	-0.09	0.03		[-0.15, -0.02]		-0.05	-2.63		**
Vegetables Per Day	0.26	0.27		[-0.28, 0.80]		0.02	0.96		.338	0.01	0.11		[-0.20, 0.23]		0.00	0.13		.894
Fruits Per Day	-0.14	0.39		[-0.89, 0.62]		-0.007	-0.35		.723	0.08	0.12		[-0.16, 0.32]		0.01	0.66		.512
Chronic Lung Disease	0.09	0.14		[-0.18, 0.36]		0.01	0.66		.509	0.00	0.04		[-0.07, 0.07]		0.00	0.08		.936
Anxiety	0.02	0.04		[-0.06, 0.10]		0.009	0.43		.668	0.01	0.02		[-0.04, 0.06]		0.010	0.51		.611
Hypertension	-0.14	0.09		[-0.31, 0.03]		-0.03	-1.59		.111	0.05	0.03		[-0.01, 0.10]		0.03	1.55		.121
Diabetes	0.15	0.14		[-0.11, 0.42]		0.02	1.13		.260	-0.01	0.05		[-0.11, 0.09]		-0.00	-0.23		.819
Depression	-0.08	0.12		[-0.31, 0.16]		-0.01	-0.64		.520	0.15	0.07		[0.01, 0.29]		0.04	2.14		**
Stroke	0.04	0.20		[-0.36, 0.44]		0.004	0.20		.842	0.14	0.06		[0.01, 0.26]		0.04	2.15		**
**Step 5**																		
*(Intercept)*	7.57	0.75		[6.10, 9.05]		0.00	10.08		***	1.40	0.29		[0.84, 1.96]		0.00	4.90		***
Sex	-0.21	0.07		[-0.36, -0.07]		-0.07	-2.89		**	0.09	0.04		[0.02, 0.17]		0.06	2.56		**
Age	-0.04	0.02		[-0.09, 0.01]		-0.04	-1.77		.077	-0.09	0.01		[-0.12, -0.07]		-0.17	-7.25		***
Residence	0.32	0.08		[0.16, 0.47]		0.09	4.11		***	-0.12	0.03		[-0.18, -0.06]		-0.08	-3.90		***
Marital Status	-0.06	0.03		[-0.11, -0.00]		-0.05	-2.10		**	-0.01	0.01		[-0.03, 0.01]		-0.02	-0.92		.360
Self Reported Memory Difficulty	0.07	0.04		[-0.01, 0.15]		0.04	1.77		.077	-0.07	0.02		[-0.11, -0.02]		-0.06	-3.04		***
Tobacco Use										-0.08	0.04		[-0.15, -0.01]		-0.05	-2.19		**
Alcohol Use	0.13	0.07		[-0.01, 0.26]		0.04	1.84		.066	-0.06	0.03		[-0.13, -0.00]		-0.04	-2.02		**
Vegetables Per Day	0.27	0.27		[-0.26, 0.81]		0.02	1.00		.317	0.01	0.11		[-0.21, 0.22]		0.00	0.07		.947
Fruits Per Day	-0.19	0.38		[-0.94, 0.57]		-0.01	-0.49		.625	0.09	0.12		[-0.15, 0.32]		0.02	0.71		.480
Chronic Lung Disease	0.07	0.14		[-0.19, 0.34]		0.01	0.53		.593	-0.01	0.04		[-0.08, 0.06]		-0.01	-0.30		.767
Anxiety	-0.00	0.04		[-0.08, 0.08]		-0.00	-0.07		.947	0.01	0.02		[-0.04, 0.05]		0.01	0.25		.801
Hypertension	-0.09	0.09		[-0.26, 0.09]		-0.02	-0.99		.324	0.05	0.03		[-0.01, 0.10]		0.03	1.62		.106
Diabetes	0.21	0.14		[-0.06, 0.48]		0.03	1.56		.120	-0.00	0.05		[-0.10, 0.09]		-0.00	-0.09		.929
Depression	-0.07	0.12		[-0.31, 0.16]		-0.01	-0.63		.531	0.16	0.07		[0.02, 0.30]		0.04	2.30		**
Stroke	0.05	0.20		[-0.34, 0.44]		0.005	0.24		.808	0.14	0.06		[0.01, 0.26]		0.04	2.20		**
Education	0.06	0.02		[0.02, 0.11]		0.06	2.63		**	0.11	0.02		[0.08, 0.14]		0.15	6.92		***
Income Quintile	0.09	0.02		[0.04, 0.13]		0.08	3.65		***	0.03	0.01		[0.01, 0.05]		0.06	2.83		**
			***South Africa***															
**Variables**			***B***		***SE***			**95% CI**				**β**		***t***			***p***	
**Step 1**																		
*(Intercept)*			1.84		0.06			[1.72, 1.96]				0.00		29.29			***	
Sex			-0.07		0.02			[-0.11, -0.02]				-0.06		-2.96			**	
Age			-0.05		0.01			[-0.07, -0.04]				-0.12		-5.85			***	
Residence			0.03		0.02			[-0.02, 0.07]				0.02		1.16			.247	
Marital Status			-0.010		0.01			[-0.03, 0.01]				-0.03		-1.23			.217	
**Step 2**																		
*(Intercept)*			2.00		0.07			[1.87, 2.13]				0.00		30.51			***	
Sex			-0.05		0.02			[-0.10, -0.01]				-0.05		-2.48			**	
Age			-0.04		0.01			[-0.06, -0.02]				-0.09		-4.18			***	
Residence			0.04		0.02			[-0.00, 0.09]				0.04		1.79			.073	
Marital Status			-0.01		0.01			[-0.02, 0.01]				-0.02		-1.13			.260	
Self-Reported Memory Difficulty			-0.11		0.01			[-0.14, -0.08]				-0.16		-7.66			***	
**Step 3**																		
*(Intercept)*			1.54		0.14			[1.26, 1.82]				0.00		10.85			***	
Sex			-0.05		0.02			[-0.10, -0.01]				-0.05		-2.39			**	
Age			-0.04		0.01			[-0.06, -0.02]				-0.09		-4.13			***	
Residence			0.04		0.02			[-0.01, 0.08]				0.03		1.75			.081	
Marital Status			-0.01		0.01			[-0.03, 0.01]				-0.03		-1.27			.205	
Self-Reported Memory Difficulty			-0.11		0.01			[-0.14, -0.09]				-0.16		-7.98			***	
Tobacco Use			-0.02		0.02			[-0.07, 0.03]				-0.02		-0.68			.498	
Alcohol Use			0.02		0.03			[-0.04, 0.07]				0.01		0.65			.519	
Vegetables Per Day			0.30		0.06			[0.18, 0.42]				0.09		4.75			***	
Fruits Per Day			0.16		0.11			[-0.06, 0.38]				0.03		1.39			.166	
**Step 4**																		
*(Intercept)*			2.13		0.25			[1.63, 2.63]				0.00		8.40			***	
Sex			-0.06		0.02			[-0.10, -0.01]				-0.05		-2.49			**	
Age			-0.04		0.01			[-0.06, -0.02]				-0.09		-4.20			***	
Residence			0.05		0.02			[0.01, 0.09]				0.04		2.17			**	
Marital Status			-0.01		0.008			[-0.03, 0.00]				-0.03		-1.41			.158	
Self-Reported Memory Difficulty			-0.12		0.01			[-0.15, -0.09]				-0.17		-8.17			***	
Tobacco Use			-0.02		0.02			[-0.07, 0.03]				-0.02		-0.75			.451	
Alcohol Use			0.02		0.03			[-0.04, 0.07]				0.01		0.61			.543	
Vegetables Per Day			0.29		0.06			[0.17, 0.41]				0.09		4.58			***	
Fruits Per Day			0.15		0.11			[-0.07, 0.37]				0.03		1.34			.181	
Chronic Lung Disease			-0.07		0.08			[-0.22, 0.09]				-0.02		-0.84			.402	
Anxiety			0.008		0.02			[-0.03, 0.04]				0.009		0.46			.644	
Hypertension			-0.01		0.02			[-0.06, 0.03]				-0.01		-0.57			.566	
Diabetes			-0.06		0.04			[-0.13, 0.01]				-0.03		-1.62			.106	
Depression			-0.11		0.05			[-0.21, -0.02]				-0.05		-2.27			**	
Stroke			-0.04		0.06			[-0.15, 0.07]				-0.01		-0.75			.456	
**Step 5**																		
*(Intercept)*			1.67		0.26			[1.17, 2.18]				0.00		6.51			***	
Sex			-0.04		0.02			[-0.09, 0.00]				-0.04		-1.89			.059	
Age			-0.04		0.01			[-0.06, -0.02]				-0.09		-4.04			***	
Residence			0.11		0.02			[0.07, 0.16]				0.10		4.70			***	
Marital Status			-0.01		0.01			[-0.02, 0.01]				-0.03		-1.19			.234	
Self-Reported Memory Difficulty			-0.09		0.01			[-0.12, -0.06]				-0.12		-5.92			***	
Tobacco Use			-0.03		0.02			[-0.08, 0.01]				-0.03		-1.38			.169	
Alcohol Use			0.01		0.03			[-0.05, 0.06]				0.005		0.21			.837	
Vegetables Per Day			0.27		0.06			[0.15, 0.40]				0.09		4.35			***	
Fruits Per Day			0.18		0.11			[-0.04, 0.40]				0.03		1.59			.111	
Chronic Lung Disease			-0.07		0.08			[-0.22, 0.08]				-0.02		-0.89			.373	
Anxiety			0.01		0.02			[-0.02, 0.04]				0.01		0.52			.604	
Hypertension			-0.01		0.02			[-0.05, 0.04]				-0.005		-0.22			.824	
Diabetes			-0.04		0.04			[-0.11, 0.03]				-0.02		-1.05			.293	
Depression			-0.09		0.05			[-0.19, 0.01]				-0.04		-1.85			.064	
Stroke			-0.04		0.06			[-0.15, 0.07]				-0.01		-0.74			.458	
Education			0.02		0.01			[0.00, 0.04]				0.05		2.28			**	
Income Quintile			0.06		0.01			[0.04, 0.07]				0.15		6.44			***	

^***^ p < .01, ** p < *.05*

## Discussion

This study examined the impact of socioeconomic status (assessed using income and education), demographics, risk factors such as lifestyle, chronic diseases and self-rated memory difficulty on adults' cognitive functioning in five selected countries via a comparative analysis of a population-based sample from the Study on Global AGEing and Adult Health Wave 1. This study found income to significantly predict cognitive functioning in all five selected countries. This indicates that, on average, a one-unit increase in one's income will increase an adult's cognitive functioning by 0.05, 0.02, 0.09 0.03, and 0.06 units for China, Ghana, India, Russia, and South Africa, respectively. This is consistent with previous studies on the role of income on the cognitive functioning of individuals [58–60]. The socioeconomic status tends to influence the development of brain structure in childhood [61], and whether or not the brain structure is formed properly as a child grows into adulthood (hippocampal formation) and even into old age could have dire consequences such as an increased risk of Alzheimer's' Disease in later life [62]. Low-income adults rarely engage in healthy lifestyles, mentally stimulating activities and may have restricted access to resources and adequate healthcare, like visiting a neuropsychologist because of low or no disposable income; this could increase the probability of cognitive decline.

The study also found education to significantly predict cognitive functioning in all the selected countries, suggesting that a one-unit increase in an individual's level of education will increase their cognitive functioning. This observation is consistent with a study by Wu et al., 2011, who found an association between a low level of education and a high risk of cognitive impairment among older adults in Taiwan [63]. Similar studies in Ghana [64], Russia [65] and South Africa [66] have noted an association between education and cognitive functioning. Research has shown that highly educated individuals tend to have greater brain reserve capacity than those with no formal education [67]. It is also believed that highly educated people are more likely to seek emotional support which can consequently result in a positive change in the function and structure of their brain [64, 68] as opposed to people with no or low level of education.

Our study found that age significantly predicted cognitive functioning in China, Ghana, Russia, and South Africa. This finding is consistent with previous studies, which suggested that age is a risk factor in cognitive decline among adults [23–25]. A study by Murman, 2015 revealed that the aging of adults tends to accelerate age-related diseases such as dementia by increasing the rate of neuronal dysfunction, neuronal loss, and cognitive decline [29]. These age-related risks also have the propensity to impair the everyday functional abilities of adults, especially in the context of developing countries. Furthermore, the type of residency (rural or urban) significantly predicted cognitive functioning in all the selected countries. A study by Yuan et al. 2020 observed that individuals residing in an urban area have better cognition [36] due to perceived better income conditions than their counterparts in rural residences. This study observed that most respondents in China, Ghana, and India lived in rural areas. Rural communities are plagued with lack of or low access to healthcare, high cost of care, a higher proportion of expenditure to income, lack of sustainable employment, and lack of access to basic social amenities. These issues tend to make rural-dwellers more susceptible to diseases. Hence, there is the need to design policies such as rolling out tailor-made insurance products, deliberate allocation of funds targeted at improving access to healthcare, and reducing the risks associated with living in these rural areas. These measures have the propensity to enhance the cognitive functioning of adults who live in rural areas. Additionally stakeholders must continue to create ecosystems that will allow individuals to venture into private businesses. They must also improve access to gainful middle-level employment opportunities for graduate adults [69], as studies have found that urban adults dwellers who are satisfied with their income conditions have improved cognitive functioning [36]. It is also essential to reduce the high cost associated with healthcare, increase access to healthcare delivery, and improve transportation systems in urban areas. It is also necessary to create avenues for exercise [70], leisure, recreation, and relaxation [71], to reduce the burden associated with urban living, as these will eventually lead to improved cognitive functioning, particularly in Russia and South Africa where the majority of respondents were urban dwellers.

Sex was observed to significantly predict cognitive functioning in China, India and Russia but not in Ghana and South Africa. This study's observed difference in cognitive functioning between males and females may be explained from two angles. First, females have a longer life expectancy than males and are likely to experience more aging-related cognitive impairments [72, 73]. Second, females in most developing nations are generally disadvantaged socially and economically compared to their male counterparts; hence they lack the needed resources to access decent healthcare and have favorable health outcomes, culminating in cognitive limitation [74].

This study observed that self-reported memory difficulty significantly predicted cognitive functioning in all the selected countries; China, Ghana, Russia, and South Africa, except India. This suggests that, on average, a one-unit increase in an individual's self-reported memory difficulty will decrease the value of cognitive functioning by 0.06 units for China and Ghana, 0.07 units for Russia, and 0.09 units for South Africa. Previous studies have shown the effect of memory difficulty on cognitive functioning [75–78]. As people age, they experience significant deficits in their memories. These deficits are primarily manifested in daily activities such as decision-making, problem-solving, and the planning of goal-directed behaviors coordinated by active manipulation, reorganization, and integrating the contents of one's memory [75]. These activities are relevant for the effective and efficient performance of these higher-level cognitive functions [75, 78]. Hence, individuals need to continue to include physical activities into their daily routines, socialize regularly, and manage stress levels to reduce the burden on their memories and consequently pre-empt cognitive decline.

Regarding the impact of lifestyle choices on adults' cognitive functioning, this study found tobacco use to significantly predict cognitive functioning in only Russia . Previous studies have revealed that people who smoke are at a higher risk of developing aging-related cognitive diseases such as all types of dementia and are at an even higher risk for Alzheimer's disease [79]. Though this study did not find tobacco use to significantly predict cognitive functioning in China, Ghana, India, and South Africa, there is the need to explore further the effect of tobacco use in these four countries. This study suspects that this disparity could be accounted for by the possible smoking status of respondents, that is, whether a smoker is currently smoking or not. The status of the smoker is pertinent because studies have shown that current smokers are at higher risk [80, 81] and experience a faster cognitive decline [82] than former smokers [83]. The study also revealed that alcohol use significantly predicted cognitive functioning in Ghana and Russia but not in China, India, and South Africa.

Fruits consumed per day were significant only in Ghana. In contrast, vegetable consumption per day was significant in predicting cognitive functioning in China and South Africa. This finding is consistent with previous studies by Chen et al. 2012 [59], who found that lower intakes of vegetables are associated with cognitive decline among elderly Chinese. Several laboratories [84, 85] and epidemiological studies [86–89] have shown that antioxidants, which are found in fruits and vegetables, are associated with cognitive function [59]. Fruits and vegetables contain vitamins C, E, folate, and carotenoids, which have been found to improve cognitive functions among higher consumers of these nutrients. Hence, individuals must introduce the habit of eating fruits and vegetables, if they wish to improve their cognitive functioning.

Finally, in terms of chronic diseases, this study did not find a significant effect of diabetes, hypertension, and chronic lung disease on cognitive functioning in all countries; meanwhile, anxiety was found to predict cognitive functioning in China and Ghana significantly. Stroke was found to significantly predict cognitive functioning in China, Ghana, and Russia, which is consistent with previous studies that have shown an association between stroke and cognitive functioning [90].

### Clinical significance and implications

Though this study found SES, some demographic characteristics, and risk factors to significantly  predict cognitive functioning, the magnitude of the effect is relatively small. For instance, the effect of SES and lifestyle on cognitive functioning was 2%, suggesting that SES and lifestyle only explained a 0.02 variability to cognitive functioning. Thus, it may appear farfetched to claim that socioeconomic status and risk factors clinically influence cognitive functioning. That notwithstanding, it should be noted that this study evaluated a large number of potential explanatory variables and that some factors (medical variables such as chronic diseases) explained even lower variance to cognitive functioning and were mostly not significant. When interpreting test results in clinical practice, neuropsychologists examine several medical and demographical factors [91, 92]. The purpose of this study was to provide insights into social factors that could influence cognitive deficiencies, so clinicians will not limit their investigation to only neurological and pharmacological considerations when assessing cognitive functioning. As a result, improving social situations may help in improving cognitive functioning. Our findings underscore the need for an interdisciplinary approach towards addressing cognitive limitations among doctors, psychologists, and social workers, ensuring more holistic healthcare to citizens of these countries.

## Conclusion

The results of this study highlight the predicting factors of cognitive functioning across all ages in developing countries. The study further analyzed the impact of SES, lifestyle, demographic characteristics and other risk factors on cognitive functioning in the context of developing countries. Based on the study findings, stakeholdersmust implement policies, encourage participation in daily physical activities, and invest in cheaper and accessible healthcare. It is also imperative to improve school enrolments for younger and middle-aged adults while strengthening adult education initiatives. These measures will help eliminate the risk factors associated with cognitive decline and improve the socioeconomic status of cohorts.

## Supplementary Information

**Additional file 1:** **Supplementary Figures **

**Additional file 2:** **Supplementary Tables **

## Data Availability

The data used for this study is the Study on Global AGEing and Adult Health (SAGE) Wave 1 conducted by the World Health Organization in six participating countries. These data are publicly available on the National Archive of Computerized Data on Aging (NACDA) website https://www.icpsr.umich.edu/web/ICPSR/studies/31381/datadocumentation.
