# Supplementary material for: Examining the impact of socioeconomic status, demographic characteristics, lifestyle and other risk factors on adults' cognitive functioning in developing countries: an analysis of five selected WHO SAGE Wave 1 Countries
Source: Int J Equity Health. 2022 Feb 25;21:31. doi: 10.1186/s12939-022-01622-7 (PMC8876754; doi:10.1186/s12939-022-01622-7)
Supplement: Supplementary file 1 — Additional file 1: Supplementary Figures [file 12939_2022_1622_MOESM1_ESM.doc]

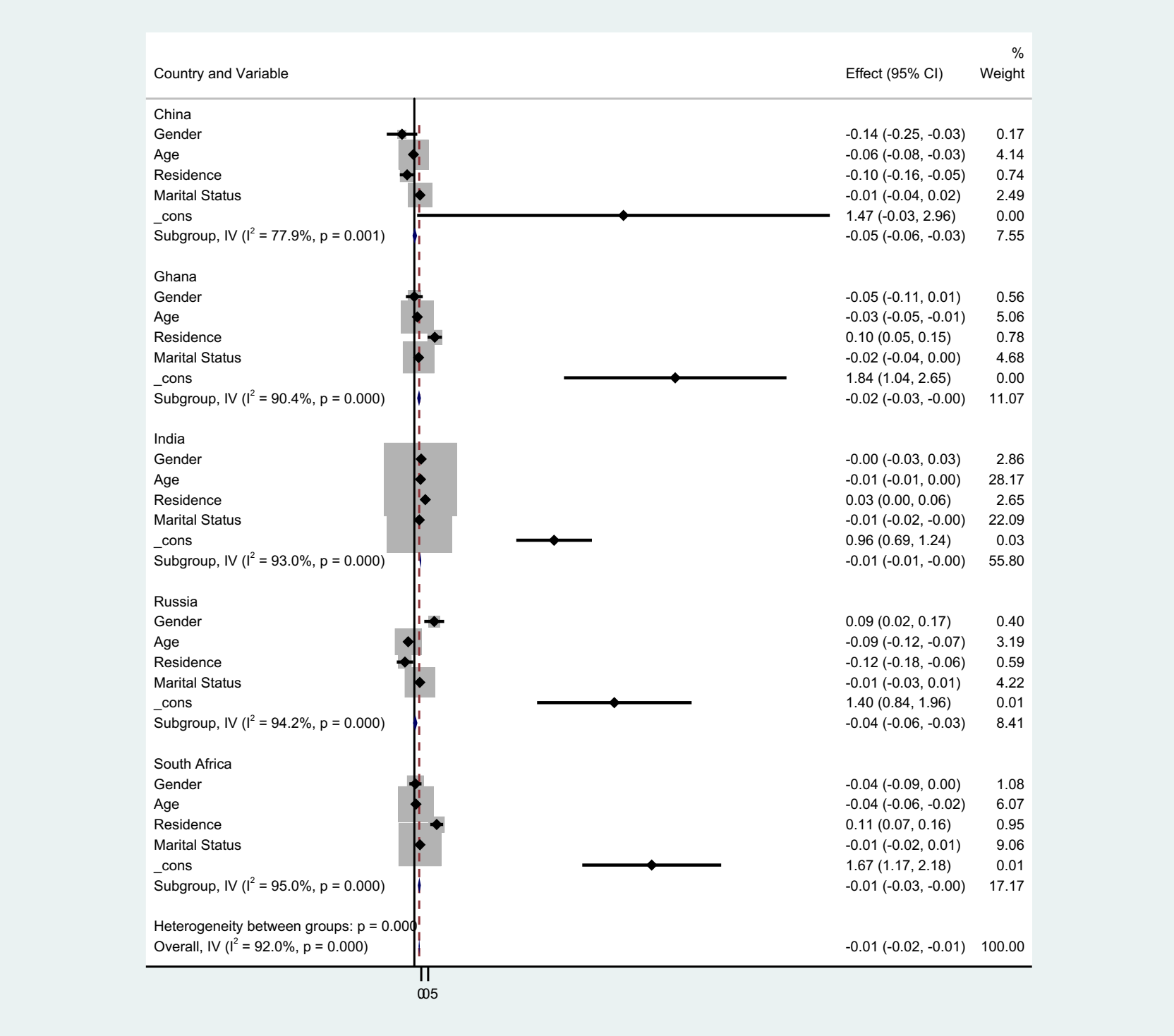
Supplementary Materials

Figure A1. Forest Plot of the Impact of Demographic Characteristics on Cognitive Functioning among the Five Selected Countries


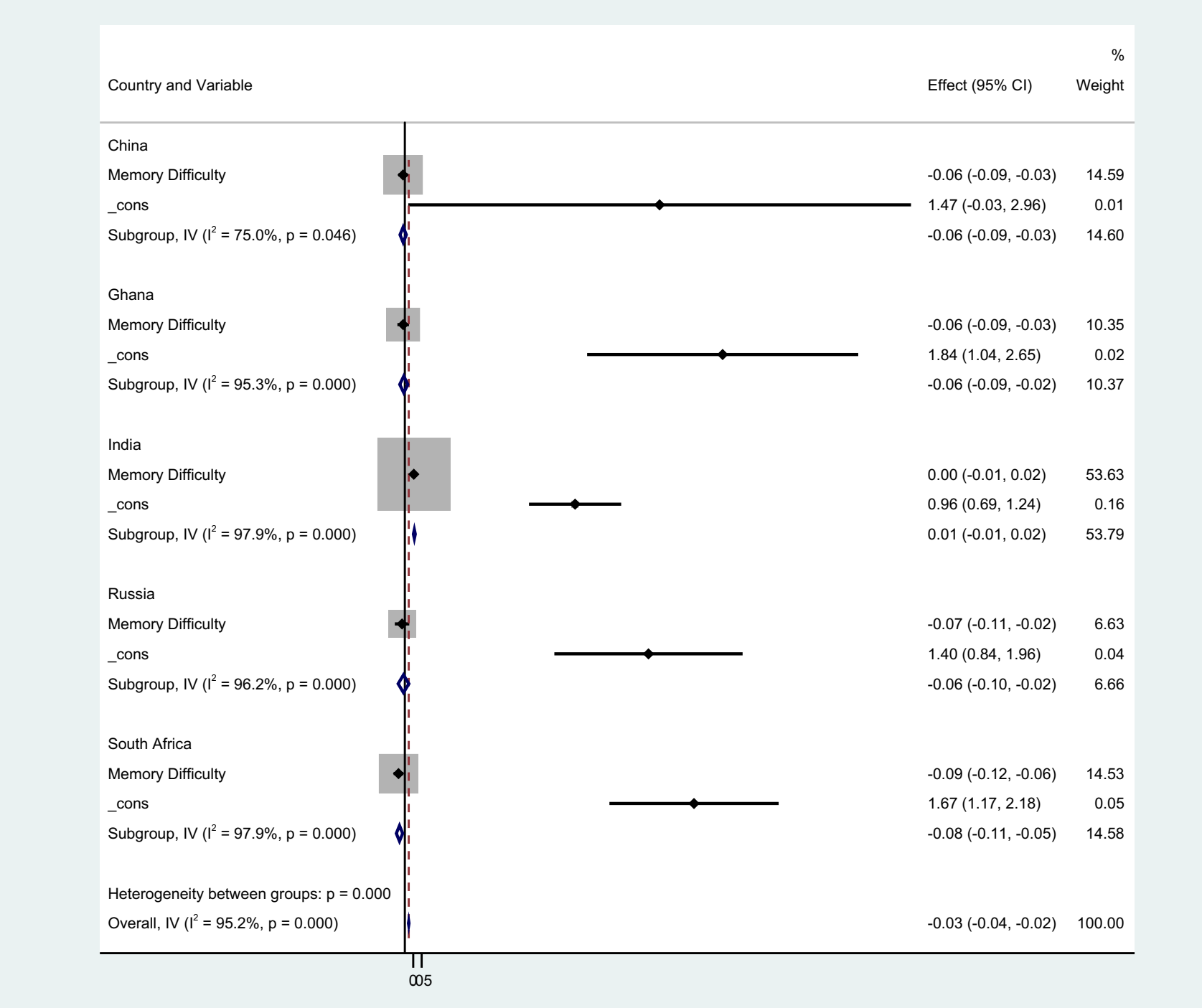


Figure A2. Forest Plot of the Impact of Self-Reported Memory Difficulty on Cognitive Functioning among the Five Selected Countries


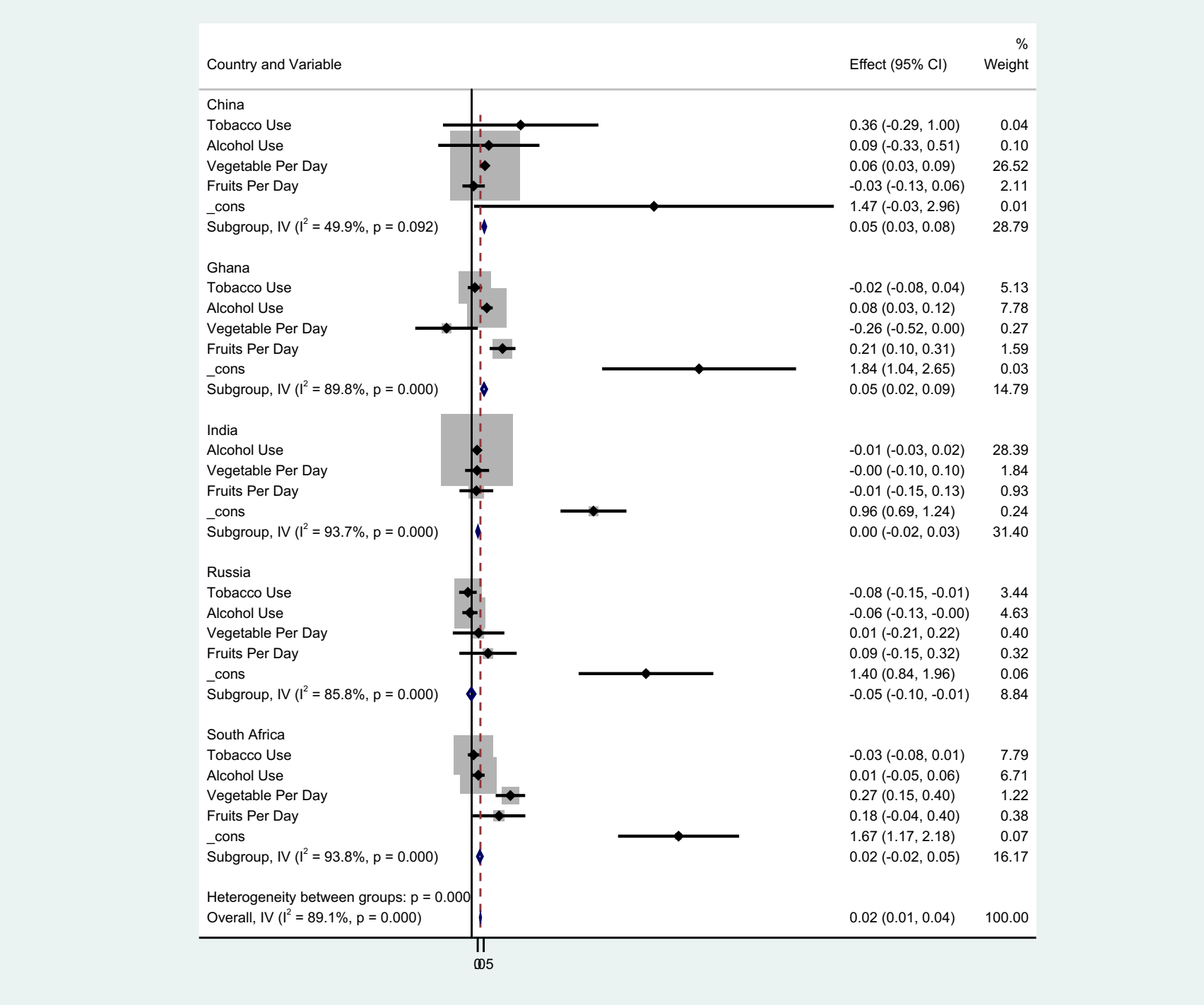


Figure A3. Forest Plot of the Impact of Lifestyle on Cognitive Functioning among the Five Selected Countries


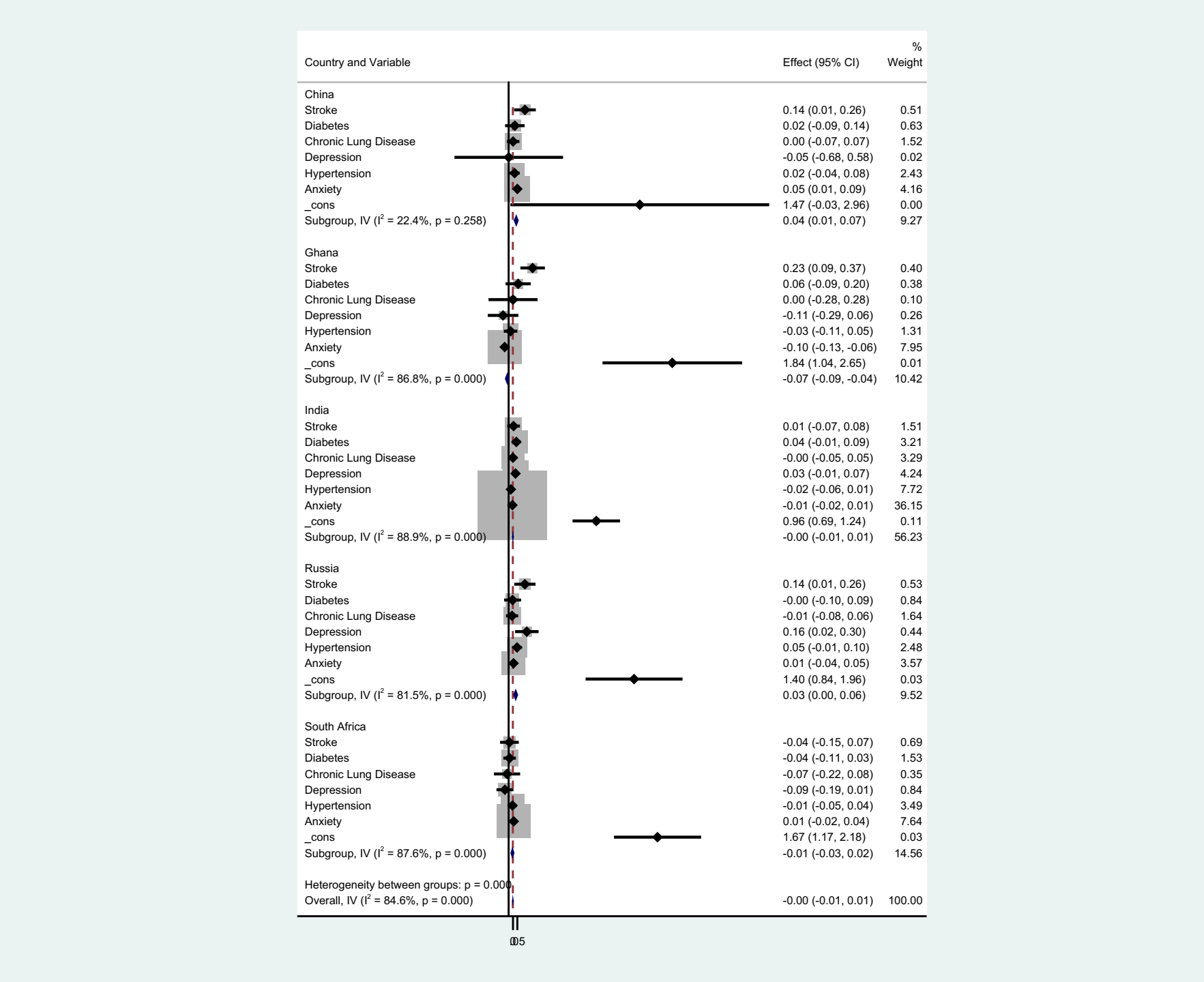


Figure A4. Forest Plot of the Impact of Chronic Diseases on Cognitive Functioning among the Five Selected Countries
