# Supplementary material for: Examining the impact of socioeconomic status, demographic characteristics, lifestyle and other risk factors on adults' cognitive functioning in developing countries: an analysis of five selected WHO SAGE Wave 1 Countries
Source: Int J Equity Health. 2022 Feb 25;21:31. doi: 10.1186/s12939-022-01622-7 (PMC8876754; doi:10.1186/s12939-022-01622-7)
Supplement: Supplementary file 2 — Additional file 2: Supplementary Tables [file 12939_2022_1622_MOESM2_ESM.doc]

Supplementary Materials

**Table A1**. Reliability Test for Items used to Assess Cognitive Functioning

| **China** | | | | | **Ghana** | | | |
| --- | --- | --- | --- | --- | --- | --- | --- | --- |
| **Item** | **Item-Test**  **Correlation** | **Item-Rest**  **Correlation** | **Average Interitem**  **Covariance** | **Alpha** | **Item-Test**  **Correlation** | **Item-Rest**  **Correlation** | **Average Interitem**  **Covariance** | **Alpha** |
| Forward Digit Span | 0.58 | 0.46 | 2.77 | 0.64 | 0.28 | 0.21 | 2.05 | 0.53 |
| Backward Digit Span | 0.57 | 0.45 | 2.84 | 0.64 | 0.29 | 0.14 | 1.98 | 0.53 |
| Immediate Recall | 0.81 | 0.42 | 1.68 | 0.78 | 0.84 | 0.33 | 0.88 | 0.65 |
| Delayed Recall | 0.79 | 0.67 | 1.83 | 0.53 | 0.74 | 0.58 | 0.85 | 0.31 |
| Verbal Fluency | 0.75 | 0.60 | 1.98 | 0.56 | 0.73 | 0.56 | 0.88 | 0.32 |
| **Test Scale** |  |  | **2.22** | **0.67** |  |  | **1.33** | **0.51** |
|  | **India** | | | | **Russia** | | | |
| Forward Digit Span | 0.53 | 0.38 | 1.01 | 0.64 | 0.62 | 0.53 | 3.42 | 0.59 |
| Backward Digit Span | 0.60 | 0.41 | 0.89 | 0.62 | 0.57 | 0.47 | 3.50 | 0.60 |
| Immediate Recall | 0.53 | 0.21 | 1.01 | 0.72 | 0.86 | 0.43 | 1.85 | 0.85 |
| Delayed Recall | 0.82 | 0.64 | 0.50 | 0.48 | 0.79 | 0.69 | 2.36 | 0.49 |
| Verbal Fluency | 0.78 | 0.55 | 0.57 | 0.54 | 0.78 | 0.68 | 2.37 | 0.49 |
| **Test Scale** |  |  | **0.80** | **0.66** |  |  | **2.70** | **0.63** |
|  | **South Africa** | | | |  |  |  |  |
| Forward Digit Span | 0.59 | 0.46 | 2.44 | 0.67 |  |  |  |  |
| Backward Digit Span | 0.67 | 0.53 | 2.19 | 0.64 |  |  |  |  |
| Immediate Recall | 0.78 | 0.42 | 1.62 | 0.77 |  |  |  |  |
| Delayed Recall | 0.76 | 0.62 | 1.77 | 0.59 |  |  |  |  |
| Verbal Fluency | 0.73 | 0.57 | 1.86 | 0.61 |  |  |  |  |
| **Test Scale** |  |  | **1.98** | **0.70** |  |  |  |  |

Table A2. Summary Statistics Table for Interval and Ratio Variables

| **China** | | | | | | | | |
| --- | --- | --- | --- | --- | --- | --- | --- | --- |
| **Variables** | ***M*** | ***SD*** | ***N*** | ***SE_M_*** | **Min** | **Max** | **Skewness** | **Kurtosis** |
| Age | 4.98 | 1.14 | 2486 | 0.02 | 1.00 | 7.00 | -0.30 | 0.36 |
| Education | 2.19 | 1.37 | 2486 | 0.03 | 0.00 | 5.00 | 0.05 | -0.83 |
| Cognitive Functioning | 1.82 | 0.59 | 2486 | 0.01 | 1.00 | 6.00 | 0.23 | 0.86 |
| Income | 3.00 | 1.37 | 2486 | 0.03 | 1.00 | 5.00 | -0.03 | -1.25 |
| **Ghana** | | | | | | | | |
| Age | 5.35 | 1.27 | 2486 | 0.03 | 1.00 | 7.00 | -0.61 | 0.17 |
| Education | 0.83 | 1.35 | 2486 | 0.03 | 0.00 | 5.00 | 1.52 | 1.03 |
| Cognitive Functioning | 1.82 | 0.59 | 2486 | 0.01 | 1.00 | 6.00 | 0.23 | 0.86 |
| Income | 2.72 | 1.35 | 2486 | 0.03 | 1.00 | 5.00 | 0.23 | -1.15 |
| **India** | | | | | | | | |
| Age | 4.70 | 1.42 | 2486 | 0.03 | 1.00 | 7.00 | -0.43 | 2.73 |
| Education | 1.10 | 1.42 | 2486 | 0.03 | 1.00 | 5.00 | 1.10 | 0.10 |
| Cognitive Functioning | 4.10 | 0.77 | 2486 | 0.01 | 1.00 | 5.00 | -0.35 | 2.26 |
| Income | 2.72 | 1.36 | 2486 | 0.03 | 1.00 | 5.00 | 0.23 | 1.82 |
| **Russia** | | | | | | | | |
| Age | 5.21 | 1.29 | 2486 | 0.03 | 1.00 | 7.00 | -0.55 | 0.16 |
| Education | 3.77 | 0.95 | 2486 | 0.02 | 0.00 | 5.00 | -1.18 | 2.04 |
| Cognitive Functioning | 1.71 | 0.71 | 2486 | 0.01 | 1.00 | 6.00 | 0.84 | 1.00 |
| Income | 3.13 | 1.43 | 2486 | 0.03 | 1.00 | 5.00 | -0.11 | -1.32 |
| **South Africa** | | | | | | | | |
| Age | 5.02 | 1.23 | 2486 | 0.02 | 1.00 | 7.00 | -0.56 | 0.77 |
| Education | 1.87 | 1.47 | 2486 | 0.03 | 0.00 | 5.00 | 0.38 | -0.77 |
| Cognitive Functioning | 1.47 | 0.54 | 2486 | 0.01 | 1.00 | 3.00 | 0.46 | -1.03 |
| Income | 3.08 | 1.40 | 2486 | 0.03 | 1.00 | 5.00 | -0.08 | -1.28 |
